# In Memoriam: George Hosfeld, M. D.

**Published:** 1884-12

**Authors:** 


### IN MEMORIAM: GEORGE HOSFELD, M. D.

PHILADELPHIA, Nov. 12th, 1884.

At a meeting of the Bœnninghausen Medical Club, of Philadelphia, the following preamble and resolutions were adopted:

Having been called upon by the will of Providence to part with our esteemed companion and trusted friend, George Hosfeld, M. D., we present the following resolutions:

1st. That during his life we recognized in him the qualities which make the honest man, the cherished friend, and the conscientious physician, and in his untimely death we bow to the Divine will which has removed from our midst one so fully qualified to adorn his chosen profession.

2d. That we extend our heartfelt sympathy to his bereaved family for the removal of a loved husband and relative, and to the community at large for the loss of a beneficent friend.

3d. That an engrossed copy of these resolutions be sent to his wife. That they be printed in the *Hahnemannian Monthly* and THE HOMŒOPATHIC PHYSICIAN, of Philadelphia, and that they be entered in the journal of this Society.

GEORGE T. PARKE, M. D., L. F. SMILEY, M. D.,
WILLIAM M. ZERNS, M. D., SAMUEL M. TRINKLE, M. D.,
GEORGE W. PARKER, M. D., DUNCAN MACFARLAN, M. D.,
F. BUCKMAN, M. D., JOSEPH HANCOCK, M. D.,
THOMAS S. DUNNING, M. D., *President*,
GEORGE W. SMITH, M. D., *Secretary*.

